# Reduction of calreticulin and ERp57 with age reveals the ER stress-related roles in cell viability and organismal lifespan regulation

**DOI:** 10.3389/fragi.2026.1758247

**Published:** 2026-02-19

**Authors:** Gracie Burdeos, Sophie Neuber-Schlicht

**Affiliations:** 1 Max Planck Institute for Biology of Ageing, Cologne, Germany; 2 Research Institute for Farm Animal Biology (FBN), Dummerstorf, Germany

**Keywords:** aging, chaperones, endoplasmic reticulum, ER stress, protein folding

## Abstract

**Introduction:**

Defects in Calreticulin (Calr) and ER protein 57 (ERp57), two tandem endoplasmic reticulum (ER) resident proteins, are associated with pathologies ranging from protein conformational disorders to impaired immune responses but are not directly linked to aging.

**Methods:**

To address this question, we analyzed Calr and ERp57 protein levels in brain sections and liver tissues from young and old mice. To evaluate the age–related reduction of Calr and ERp57 in vivo and its physiological implications, lifespan and ER-stress assays were conducted using C. elegans strains. Subsequently, transient knockdown and overexpression of Calr and ERp57 were performed in N2a cells, followed by assessments of cell viability, protein aggregation, apoptotic pathways, and epistasis under both basal and stress conditions.

**Results:**

Here, we report that Calr and ERp57 expression is ubiquitously decreased with age in mouse tissues, and RNAi-mediated inhibition of their homologs in C. elegans leads to ER stress–related lifespan shortening. Knockdown of Calr and ERp57 in N2a cells reduces cellular viability by exacerbating protein aggregation, ER stress, and activation of pro–apoptotic pathways. In contrast, overexpression of Calr and/or ERp57 in N2a cells results in a robust increase in stress tolerance, cell viability, and suppression of apoptotic signaling pathways.

**Conclusion:**

Taken together, our findings suggest that the age-related reduction of Calr and ERp57 may serve as a potential pro-aging biomarker, contributing to the disruption of ER homeostasis and affecting cell survival and organismal lifespan.

## 1 Introduction

Appropriate folding and post-translational modifications are essential steps that occur immediately after nascent polypeptides are synthesized and released from the ribosomal machinery, ensuring the functional quality of nearly all proteins, whether within the cells or in their plasma membranes (Balch et al., 2008; Hutt et al., 2009). The endoplasmic reticulum (ER), a subcellular compartment, plays a crucial role in many cellular processes, including cellular calcium ion homeostasis and lipid synthesis, particularly protein folding and post-translational modifications, along with ER-associated degradation (ERAD) and unfolded protein response systems. ER residential proteins, such as molecular chaperones, are, thus, extremely important in assisting protein conformational folding in the cells under physiological or stress-related pathological conditions along proper pathways (Michalak et al., 2009; Koga et al., 2011). Calreticulin (Calr) and ER protein 57 (ERp57) are molecular chaperones among the group of ER resident proteins working in tandem to generate a specific molecular chaperoning activity to facilitate proper folding of newly translocated delicate nascent polypeptides. Specifically, Calr ensures the correct attachment of amino sugar classes to the monoglucosylated carbohydrate of a newly synthesized polypeptide in its attempt to form its folding structure (Mesaeli et al., 1999; Michalak et al., 1999; Nakamura et al., 2001; Caramelo and Parodi, 2015), whereas ERp57 catalyzes the rearrangement of the disulfide bond within the protein substrate to signal protein maturation and proper folding (Zapun et al., 1998). All these complex procedures collectively ensure the production of well-folded, functional proteins that are ready to be delivered by secretory pathways to the appropriate locations, where they fulfill their assigned functions within cells. Although the ER has an army of residential chaperones, including Calr and ERP57, that act as the “checkers and removers” safeguarding the protein folding process, a considerable proportion of translocated proteins within the ER is still inevitably improperly folded in various physiological/pathological circumstances due to a range of reasons. One of the critical issues, unsurprisingly, is because of the safeguards—“checkers and removers.” The perturbation of Calr and ERp57 in cells has been associated with protein conformational disorders, such as neurodegenerative diseases, metabolic disorders, myopathies, liver diseases, and a specific type of systemic disorder, amyloidosis (Esser et al., 2004). A well-documented and prominent function of Calr and ERp57 has been reported for their crucial roles in immune response. Calr and ERP57 physically interact with MHC class-I molecules and form the peptide-loading complex with additional associated factors in the antigen presentation process and the assembly of CD1d molecules (Dong et al., 2009; Kang and Cresswell, 2002), and the failure of these two ER proteins to function in tandem results in impaired immune surveillance and responses (Panaretakis et al., 2008).

In addition to the fact that disruption of Calr and ERp57 machinery triggers catastrophic pathologies, as mentioned earlier, there is no study that demonstrates/elucidates the mechanism of such disruption in the aging process. Rationally, the feature of ER-associated protein folding perturbation with age leads to a reduced ability to detect and eliminate misfolded proteins, leading to the accumulation of these misfolded proteins, particularly in old cells, organs, or organisms. Importantly, long-term high levels of compromised proteins are an indicator of increased damaging agents with age (Koga et al., 2011). These scenarios provide tangible evidence that further understanding of ER chaperones, such as Calr and ERp57, and their specific functions in aging is both interesting and important. In this study, we intended to determine the underlying roles and mechanisms of ER residential proteins Calr and ERp57 in cellular survival and organismal aging. We found a ubiquitous and robust reduction of Calr and ERp57 protein expression with aging in mouse tissue. Genetic manipulations of Calr and ERp57 expression significantly affect cell viability and organismal lifespan through ER-stress-related mechanisms. Our novel discovery demonstrates, for the first time, that the reduction of Calr and ERp57 may play an important role in cell death and organismal aging.

## 2 Materials and methods

### 2.1 Mouse tissue preparation and protein extraction

Female C57BL/6 mice aged 12 weeks (young) and 104 weeks (old) were sacrificed, and then tissues from the brain regions and liver were dissected and snap-frozen for later use. Pieces of each tissue were homogenized using RIPA buffer and further sonicated for 10 s. Samples were briefly vortexed and centrifuged at 10,000 *g* at 4 ^O^C for 10 min. The supernatant was collected, and protein concentration was measured using a BCA kit (Thermo Fisher Scientific, Waltham, MA, United States), following the manufacturer’s protocol. Animal experiments were carried out in accordance with EU directive 2010/63/EU for animal experiments and the German Animal Welfare Act (TierSchG, 2006) and were approved by the regional authorities (LANUV NRW, approval number: AZ 84-02.05.40.14.133).

### 2.2 *C. elegans* strains

The *Caenorhabditis elegans* WT strain N2 was a gift from the Reinke laboratory. The strains CB1370 [daf-2 (e1370) III], CF1380 [daf-16 (mu86) I], and SJ4005 (zcIs4 V) were purchased from the *Caenorhabditis* Genetics Center (CGC, University of Minnesota, United States). All the strains were maintained on *E. coli* OP50, as previously described (Qian et al., 2015a).

### 2.3 RNA interference

The RNAi clones *crt-1* (V-4H05) and *pdi-3* (I-3M18) were acquired from GenomeCUBE at Source BioScience (Nottingham, United Kingdom). Feeding bacteria HT115 containing the empty vector L4440 were used as food for the control group. RNAi-induction was performed by feeding the worms with bacteria that produced dsRNA against the gene of interest. In brief, the RNAi clone in *E. coli* was incubated overnight at room temperature on RNAi agar plates with 25 μg/mL carbenicillin and 1 mM isopropyl thiogalactoside (IPTG) (Tocris Bioscience, Bristol, United Kingdom) to induce dsRNA expression on day 1. On day 2, L4 larvae were transferred to the seeded plates to be monitored for their lifespans. WT (CGC), CB1370 (daf-2 mutant, CGC), and CF 1380 (daf-16 mutant, CGC) worms were subjected to RNAi treatment.

### 2.4 Examination of the ER stress in sensor worms

Strain SJ4005 (ER-stress sensor, CGC) was used to study GFP expression following RNAi induction. In brief, the ER stress reporter strain SJ4005 expresses hsp-4:GFP, where *hsp-4* is the *C. elegans* ortholog of the ER chaperone BiP/GRP78 and is transcriptionally induced by the activation of the unfolded protein response (Kapulkin et al., 2005). L4 larvae were transferred to the seeded RNAi plates to be monitored for at least 72 h. Then, the images of the worm were recorded using a CCD camera mounted on a fluorescence dissecting microscope (Olympus SZX16). ImageJ software was used to quantify the GFP intensity. Specifically, ImageJ was used to measure the integrated fluorescence density of the area of interest and the background fluorescence. The corrected total worm fluorescence = integrated density of the area of interest − background fluorescence.

### 2.5 Lifespan assays

To quantify lifespan, L4 larvae from the age-synchronized worm populations were transferred to NGM plates containing 100 ug/mL ampicillin and 500 nM 5-fluoro-20-deoxyuridine (FUDR) (Thermo Fisher Scientific), seeded with sufficient OP50 bacteria. Worms were monitored by tapping their head with a platinum worm pick every 1 or 2 days until they were dead. Worms were scored as dead if they did not respond to tapping by moving their heads. Worms that had fled or crawled off the agar and died on the side were censored and removed from analysis. A minimum of three individual experiments were performed for each group. Log-rank test was applied for statistical analysis.

### 2.6 Cell culture

The mouse neuro 2a cell line (N2a) was procured from Sigma (St. Louis, MO, United States). The cells were cultured in Dulbecco’s modified Eagle’s medium (DMEM) (Sigma) containing 0.3 g L^−1^ L-glutamine and 2.0 g L^−1^ sodium bicarbonate supplemented with 10% fetal bovine serum (FBS; Biowest, France), 100 kU L^−1^ penicillin, and 100 mg L^−1^ streptomycin (Gibco, Carlsbad, CA, United States) at 37 °C in a 5% CO_2_/95% air atmosphere in a humidified incubator.

### 2.7 RNAi and overexpression plasmid preparation

RNAi plasmids against mouse Calr and ERp57 were cloned and constructed according to the protocol of the BLOCK-iT™ Pol II miR RNAi Expression Vector Kit (pcDNA™6.2-GW/EmGFP-miR vector, K4936-00, Invitrogen, Carlsbad, CA, United States). In brief, target-specific oligonucleotides were designed using the BLOCK-iT RNAi designer, annealed, and cloned into the miRNA expression cassette of the pcDNA™6.2-GW/EmGFP-miR vector. Recombinant plasmids were propagated in *E. coli*, and correct insertion was confirmed by DNA sequencing (Eurofins, Ebersberg, Germany). Validated RNAi constructs were used for subsequent cell-based experiments. Plasmids containing Calr (pCMV6 entry, c-Myc tagged-Calr) and ERp57 (pCMV6 entry, c-Myc tagged-PDIA3) were procured from OriGene (Rockville, MD, United States). All plasmid DNAs were prepared using an Endo-Free Plasmid Maxi Kit (QIAGEN, Hilden, Germany) for the experiments.

### 2.8 Plasmid DNA transfection and cell imaging

N2a cells (0.3 × 10^6^ cells per well) were pre-incubated with 10% FBS/DMEM in 6-well culture plates for 24 h. Transient transfections were performed with Lipofectamine 2000 (Invitrogen), according to the manufacturer’s protocol. In brief, 2.5 μg of plasmid DNA and 5 µL of Lipofectamine 2000 were used per transfection. After incubation for 24 h, the medium was replaced with fresh DMEM and further incubated until 80% transfection efficiency was observed (72 h post-transfection). The transfection efficiency and cell morphologies were determined using an automated inverted microscope (Leica DMI4000 B, Leica Microsystems, Wetzlar, Germany). Total RNAs and proteins were extracted approximately 72 h post-transfection.

### 2.9 Cell viability and cellular stress assays

For cell viability assays, 48 h post-transfection, N2a cells (5,000 cells per well) were seeded and pre-incubated with 10% FBS/DMEM in 96-well culture plates (Thermo Fisher Scientific). After incubation for 24 h (72 h post-transfection), the number of viable cells was determined using WST-1 reagent, according to the manufacturer’s instructions (Dojindo Laboratories, Kumamoto, Japan). In brief, WST-1 reagent (10 μL) was added to the medium and incubated at 37 °C for 3 h. Absorbance (450/655 nm) of the medium was measured using a microplate reader (Model 550, Bio-Rad Laboratories). In parallel with the cell viability assay, separate N2a cells, 48 h post-transfection, were plated in 96-well culture plates and further incubated for 24 h. After 24 h (72 h post-transfection), cells were washed with phosphate-buffered saline (PBS), and the medium was replaced with 100 µM tunicamycin (Thermo Fisher Scientific) and was further incubated for 24 h (stress assay). The number of viable cells was determined using a WST-1 kit.

### 2.10 Protein aggregation assay

Transfected cells were washed with PBS, scraped, and transferred into a 1.5 mL tube, followed by centrifugation at 400 *g* for 5 min. The supernatant was removed, and the cell pellet was re-suspended with 200 μL of PBS, followed by pipetting to avoid cell clumping. Cell suspensions were then fixed with 4% formaldehyde for 30 min at RT. Fixed cells were centrifuged at 800 *g* for 15 min, washed with PBS twice, and then permeabilized (0.5% Triton X-100, 3 mM EDTA, pH 8) for 30 min on ice. Cells were then centrifuged at 800 *g* for 15 min, washed twice with PBS, and then incubated using the ProteoStat^®^ Aggresome Detection Kit (ENZO, Farmingdale, NY, United States). Fluorescence (Ex: 550/Em: 600 nm) of the medium was measured using a microplate reader (Bio-Rad).

### 2.11 Isolation of total RNA and analysis of mRNA expression

Total RNA from selected mouse tissues and harvested cells was isolated using the TRIzol method and an RNeasy kit (QIAGEN) for real-time quantitative reverse transcription-polymerase chain reaction (RT-PCR). cDNA was synthesized using SuperScript III (Thermo Fisher Scientific), and PCR amplification was performed using a Veriti Thermal Cycler (Thermo Fisher Scientific) and 7900HT Fast Real-Time PCR Detection System (Applied Biosystems, Foster City, CA, United States) using SYBR Premix Ex Taq (Applied Biosystems) and gene-specific primers for Calr (forward 5′-AGA​CCC​TGC​CAT​CTA​TTT​CAA​AG-3′, reverse 5′-CAG​CCC​TTT​ATC​CTT​CTC​CAG-3), ERp57 (forward 5′-GAG​GCT​TGC​CCC​TGA​GTA​TG-3′, reverse 5′-GTTGGCAGTGCAATCCACC-3′), spliced X-box binding protein 1 (spXBP1) (forward 5′-TGC​TGA​GTC​CGC​AGC​AGG​TG-3′, reverse 5′-GCT​GGC​AGG​CTC​TGG​GGA​AG-3′), and beta actin (β-actin) (Eurofins, Ebersberg, Germany). PCR conditions were 95 °C for 60 s, 95 °C for 5 s, and 60 °C for 30 s for over 40 cycles. Total RNA isolation from worms and qPCR were performed as previously described (Qian et al., 2015b). In brief, total RNA was isolated from 10 adult worms per sample using the RNeasy Mini Kit (QIAGEN). Worms were washed thrice in M9 buffer, and then the samples were re-suspended in 350 μL lysis buffer with β-mercaptoethanol (Sigma) mixed with an equal volume of 70% ethanol. The mixture was transferred to a spin column and was followed by washing steps, according to the manufacturer’s protocol. DNase digestion was performed in the column, and RNA was eluted with RNase-free water. A total of 12 μL RNA was used to synthesize cDNA using the Omniscript Kit (QIAGEN). For real-time PCR, each 25 μL reaction containing 12.5 μL of 2x SYBR Green Supermix (Bio-Rad), 0.4 μM of each gene-specific primers for crt-1 (forward 5′-CTG​GGA​TGA​CGA​GAT​GGA​CG-3′, reverse 5′-TCGTTCCTGACTTGACCTGC-3), pdi-3 (forward 5′-ACA​ATG​GAG​GAC​GCG​AAG​TT-3, reverse 5′-ACG​CCA​TAA​TGA​AGG​CAC​CA-3′), and 2 μL of template cDNA was performed on a C1600 Thermal Cycler (Bio-Rad), following the above-mentioned PCR conditions. The relative gene expression level was normalized to act-1 and calculated using the ΔΔCt (cycle threshold) method.

### 2.12 Simplified epistasis assay

Combined transient transfections were performed in pre-incubated N2a cells in 6-well culture plates (Thermo Fisher Scientific). In brief, knockdown (KD) and overexpression (OE) plasmid DNAs for both Calr and ERp57 were co-transfected separately, and OE Calr/KD ERp57 and OE ERp57/KD Calr plasmid combinations were also co-transfected. Lipofectamine 2000 (Invitrogen) was used as the transfection vehicle following the manufacturer’s suggestions. After overnight incubation with the transfection mix, the medium was replaced with fresh 10% FBS/DMEM. Co-transfected N2a cells were then seeded in 96- and 6-well plates and further incubated for an additional 72 h. The cells seeded in 96-well plates were used for the cellular viability assay as described earlier, and the remainder was used for Western blotting analysis.

### 2.13 Immunoprecipitation assay

Cells overexpressing Calr were subjected to immunoprecipitation assay using the Pierce Magnetic c-Myc-Tag IP/Co-IP Kit (Thermo Fisher Scientific), following the manufacturer’s protocol. In brief, cells were washed with ice-cold PBS, and appropriate Mag c-Myc IP/Co-IP Buffer-1 was added, followed by centrifugation at ∼13,000 x *g* for 10 min. The supernatant was transferred to a new tube for protein concentration analysis using the BCA protein concentration kit (Thermo Fisher Scientific). An appropriate amount of Pierce Anti-c-Myc magnetic beads were added to a 1.5-mL micro-centrifuge tube and washed with 175 µL of buffer 1, followed by the collection of the beads using a magnetic stand. The beads were further washed by adding 1 mL of buffer 1 with gentle vortexing for 1 min. Samples were then added onto the pre-washed beads and incubated at room temperature for 30 min using a rotating shaker. The beads were then washed twice with diluted Mag c-Myc IP/Co-IP buffer 2, with gentle mixing. After the beads were collected, 300 µL of ultra-pure water was added with gentle mixing, followed by the collection of beads using a magnetic stand. The beads were re-suspended in 100 µL of 1x non-reducing sample buffer with brief mixing and then incubated at 95 °C for 5 min in a heat block. Samples were then subjected to SDS-PAGE and Western blot analysis.

### 2.14 Western blot analysis

Proteins from mouse tissues and N2a cell lysates were separated by SDS-PAGE using Any kD Criterion TGX Stain-Free Gel (Bio-Rad). The protein bands were transferred to a nitrocellulose membrane (GE Healthcare). After blocking for 1 h, the membranes were incubated with primary antibodies for Calr, ERp57, C/EBP-homologous protein (CHOP), cleaved caspase-3, phospho-translation initiation factor (*p*-eIF2α), and β-actin (Cell Signaling Technology, Danvers, MA, United States) and further incubated overnight, followed by horseradish peroxidase-conjugated secondary antibody (Cell Signaling Technology). ECL Prime (GE Healthcare, Chicago, IL, United States) was used for detection. Band intensities were measured using Image Lab software version 3.0 (Bio-Rad).

### 2.15 Data analysis

GraphPad Prism 10.2.3 (GraphPad Software, San Diego, CA, United States) was used for statistical analysis. Data are expressed as the mean ± standard deviation (SD). One-way ANOVA was performed, followed by the Bonferroni/Dunn test for multiple comparisons. Differences were considered significant at *p* < 0.05.

## 3 Results

### 3.1 Calr and ERp57—potential biomarkers for tissue aging

**FIGURE 1:**
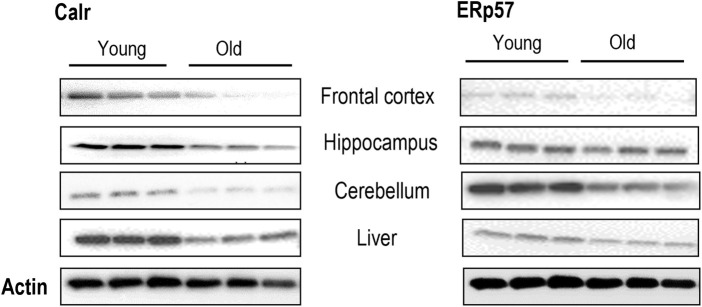
Expression of Calr and ERp57 is significantly reduced with age in mouse tissues. Western blots of Calr and ERp57 in the brain regions and liver tissues of young and old mice. Each Western blot is a representative example of data from three replicate experiments. Data are expressed as the mean ± SD (n = 3). Means with the symbol (*) differ significantly; *p* < 0.05.

Biomarkers of aging are valuable tools for quantifying physiological age, evaluating the health status, and potentially predicting the lifespan and healthspan in humans. We extensively examined the protein expression of Calr and ERp57 in multiple tissues, and the results showed a ubiquitous reduction in protein levels in mouse brain regions and liver tissues with advancing age (Figure 1A; Supplementary Figure 1). This indicates that the decline of Calr and ERp57 with age is negatively correlated. This marked negative correlation of Calr and ERp57 expression with age may lead to intriguing possibilities. First, they could serve as novel biomarkers of tissue aging; they could also play crucial roles in the aging process, similar to Klotho, Trip13, and Bmk1 (Kurosu et al., 2005; Qian et al., 2015a; Qian et al., 2015b). This raises an array of critical questions, such as whether the age-related reduction of Calr and Erp57 is just a biomarker for tissue aging or may have a prominent biological impact on the aging process.

### 3.2 Inhibition of the expression of Calr and/or ERp57 by RNAi in worms shortens their lifespans

**FIGURE 2:**
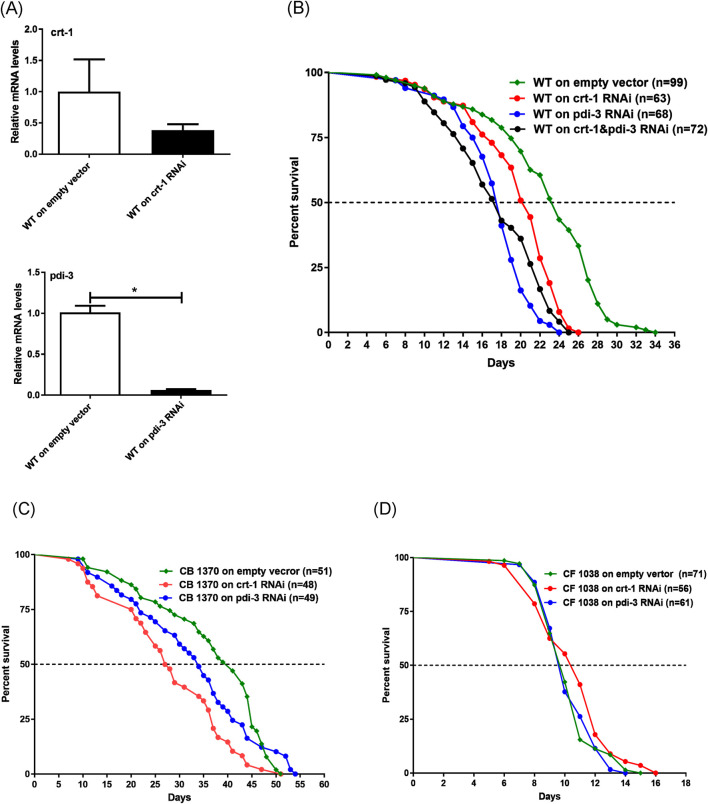
Inhibition of *crt-1* and *pdi-3* expression by RNAi shortened worm lifespan. **(A)** Relative abundance of N2 worm *crt-1* and *pdi-3* mRNA levels after *crt-1* and *pdi-3* RNAi treatment, respectively. **(B)** Both the median (13%, 25%, and 25%) and the maximum (24%, 29%, and 26%) lifespan were shortened by *crt-1*, *pdi-3*, and *crt-1* + *pdi-3* RNAi (n = 63, 68, and 72) compared to the empty vector (n = 99) (log-rank test, *p* < 0.0001). **(C)** Median lifespan was shortened (33% and 17%) by *crt-1* and *pdi-3* RNAi (n = 48 and 49) in CB1370 (*daf-2* mutant) worm compared to that in worms with empty vector (n = 51), though the maximum lifespan was not impacted. **(D)** Neither the median nor the maximum lifespan was impacted by *crt-1* and *pdi-3* RNAi compared to that in empty vector in the CF1038 (*daf-16* mutant) worm strain.

To test the hypothesis that the reduction of Calr and ERp57 significantly impacts the aging process, *C. elegans*, a well-characterized model organism with a relatively short lifespan, was utilized to examine their effects on animal longevity and potential underlying mechanisms (Qian et al., 2015a). Inhibition of worm *crt-1* (*Calr* equivalent) and *pdi-3* (*ERp57* equivalent) by RNAi was applied first to both the wild-type (WT) and long-lived Daf-2 mutant worm strains for lifespan measurement. First, we assessed the effects of RNAi on the expression of both *crt-1*, which showed an approximately 65% decrease, and *pdi-3*, which showed over a 90% decrease, using quantitative reverse transcription polymerase reaction (qRT-PCR) (Figure 2A). After confirming the inhibition of *crt-1* and *pdi-3* expression, we subjected *crt-1* and *pdi-3* RNAi-treated worms and WT worms to lifespan measurement. The median lifespans of WT, *Crt-1*, and *Pdi-3* RNAi worms were 23 days, 19.5 days, and 17 days, respectively, with the maximum lifespans of 34 days, 26 days, and 24 days, respectively (Figure 2B). The results indicated, as anticipated, that decreasing the expression of *crt-1* or *pdi-3* in worms reduced both the median and maximum lifespan up to 25% and 29%, respectively, compared to those in the WT (log-rank test, *p* < 0.001) (Figures 2A,B). Moreover, a marked lifespan reduction of approximately 25% for both median and maximum lifespans was further observed in the *crt-1* + *pdi-3* RNAi co-treatment (Figure 2B). Next, we tested the RNAi of *crt-1* and *pdi-3* in long-lived Daf-2 mutant *C. elegans* (CB1370), a documented strain with known lifespan extension through insulin pathway regulation (Kenyon et al., 1993). A significant median lifespan reduction was observed with *crt-1* and *pdi-3* RNAi treatment (33% and 17%, respectively) in Daf-2 mutant worms (Figure 2C). It indicates that *Crt-1* and *Pdi-3* affect the insulin receptor-regulated longevity pathway. We further examined the effect of *crt-1* and *pdi-3* inhibition on short-lived Daf-16/FOXO mutant worms (CF1038), a key downstream component of the insulin pathway in lifespan regulation, but no additional impact on lifespan was detected (Figure 2D). This suggests that *crt-1* and *pdi-3* may interact with the signaling mediators between the insulin receptor (DAF-2) and the transcriptional activator FOXO (DAF-16) in regulating worm longevity. Although prior proteomics studies in *C. elegans* support age-associated deterioration of ER homeostasis, aligning with our observation that reduced *crt-1* and *pdi-3* function compromises survival during aging (Walther et al., 2015; Klang et al., 2014), our findings established a novel role for *crt-1* and *pdi-3* expression in worm lifespan regulation.

### 3.3 RNAi inhibition of Calr and ERp57 triggers ER-stress-related lifespan shortening in worms

**FIGURE 3:**
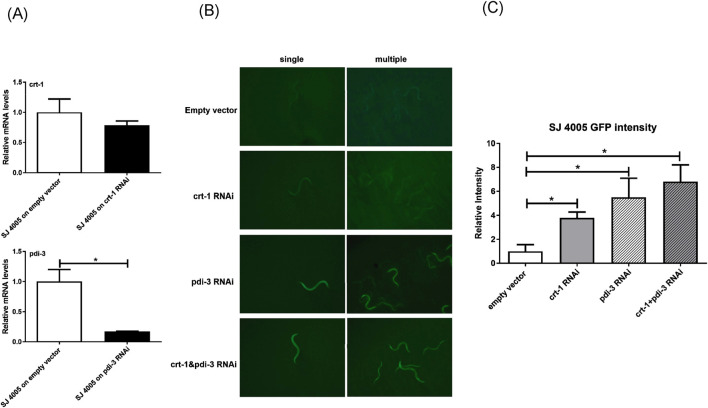
Inhibition of *crt-1* and *pdi-3* exacerbated ER stress in worms. **(A)** Relative abundance of *crt-1* and *pdi-3* mRNA levels in SJ4005 worms after *crt-1* and *pdi-3* RNAi treatment, respectively. **(B)** Green fluorescent signals in SJ4005 worms were increased by *crt-1*, *pdi-3*, and *crt-1*+*pdi-3* RNAi. **(C)** GFP intensity (indicator of ER stress) was increased by 74%, 82%, and 85% by *crt-1*, *pdi-3*, and *crt-1*+*pdi-3* RNAi, respectively, compared to that in the empty vector worms (*p < 0.005, n = 3).

ER-stress and aging intricately interact with each other in the process of aging and the incidence of age-related chronic diseases (Macario and Conway, 2002). To further elucidate the mechanism of lifespan regulation by *crt-1* and *pdi-3* in worms, the decrease of *crt-1* and *pdi-3* in worms may likely have a direct impact on the ER homeostasis since both Calr and ERp57 are ER resident chaperones (Figure 3A). We thus utilized a worm strain equipped with an ER-stress sensor, the GFP transgene SJ4005 worm, to test this idea (Bennett et al., 2014). *crt-1* or *pdi-3* RNAi treatment in SJ4005 worms resulted in a 3- to 5-fold increase in ER stress, as measured worm GFP intensity, and further induction of ER stress was evident upon *crt-1* and *pdi-3* RNAi co-treatment (Figures 3B,C). Taken together, the observed heightened ER stress upon *crt-1* and *pdi-3* depletion might contribute to the reduced lifespan of *C. elegans*.

### 3.4 Knockdown of Calr and ERp57 reduced the cellular viability

**FIGURE 4:**
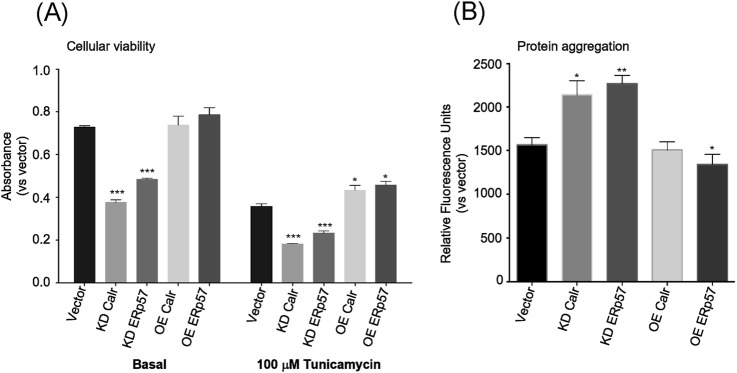
Calr and ERp57 regulate the cellular viability and protein aggregation in N2a cells. **(A)** Cellular viability analysis of Calr and ERp57 RNAi or overexpression cells under basal (left side) and stress-induced (right side, +100 µM tunicamycin) conditions. **(B)** Protein aggregation levels in cells treated with either Calr or ERp57 RNAi or overexpression measured using the ProteoStat Protein Aggregation Kit at Ex: 550 nm and Em: 600 nm. Data are expressed as the mean ± SD (n = 6). Means with the symbol (*) differ significantly; *p* < 0.05.

To further dissect the molecular mechanism of Calr and ERp57 in cellular/tissue/organismal aging, a neuronal cell line N2a was utilized in the study. First, cellular viability was measured, and it was significantly affected by the corresponding reductions of Calr and ERp57 via RNAi in N2a cells, which indicates that these two proteins are essential for cellular proliferation/survival (Figure 4A, left; Supplementary Figures 2A-E). Given that the reduction of Calr and ERp57 shortens worm lifespan through ER homeostasis (Figures 2B–D), we assumed that the knockdown of Calr and ERp57 in N2a cells would likely decrease cellular viability by compromising their ER-stress-handling capacity. To test this idea further, ER stressor tunicamycin, a known substance that impairs ER homeostasis, was applied in the cell study. As expected, knockdown of Calr and ERp57 proteins in N2a cells resulted in a further marked significant decrease (≈40%) in cell viability (Figure 4A, right). This demonstrated that the reduction of these two ER proteins rendered the cells more vulnerable to ER stress. ER chaperones participate in the cellular proliferation process by providing efficiently folded proteins that are necessary for cellular proliferation and development; therefore, reduced Calr and ERp57 expressions might lead to the loss of properly folded protein transport, thereby activating ER-stress-induced cell death via apoptotic signaling cascade. We then sought to determine whether overexpression of Calr and ERp57 has a beneficial biological effect *in vitro* under basal and stress conditions. Overexpression of Calr and ERp57 proteins showed a slight increase in cellular viability at the basal level (Figure 4A, left; Supplementary Figures 2 A-C, F-H). Interestingly, under stress conditions, cells overexpressing Calr and ERp57 showed a substantial increase in cellular viability compared to their basal counterparts (Figure 4A, right). It indicates that Calr and ERp57 play crucial roles in ER-stress response. The observed stress resistance of cells overexpressing Calr and ERp57 indicate that these two proteins could be a possible mediator for ER induced-cellular stress via the restoration of ER homeostasis.

### 3.5 Protein aggregation is associated with Calr and ERp57 reduction in N2a cells

Misfolded proteins normally form aggregates in the ER lumen that, when left unprocessed, accumulate and induce ER stress. We then examined whether the significant reduction in cellular viability observed in Calr and ERp57 knockdown N2a cells could be mediated by increased protein aggregation. To address this question, we measured protein aggregation in Calr and ERp57 knockdown cells calorimetrically using the ProteoStat^®^ Aggregation Kit. A significant accumulation of aggregated proteins was observed in Calr and ERp57 knockdown cells, whereas the overexpressing counterparts showed a marked reduction, except for Calr overexpression, which exhibited a level similar to that of the control (Figure 4B). Protein aggregations have been implicated as the consequence of perturbed ER protein folding machinery. Disturbances in this protein folding process orchestrate an array of cellular phenotypes, such as cell death via apoptosis. Our observation of increased protein aggregates in the knockdown cells confirmed that the reduction of ER key chaperones such as Calr and ERp57 can cripple the ER protein folding system, thus leading to the accumulation of misfolded proteins. These data are in agreement with the previous reports regarding the key role of chaperones in the protein folding machinery (Koga et al., 2011).

### 3.6 Calr and ERp57 reduction prompts heightened ER-stress-associated signaling response and apoptosis

**FIGURE 5:**
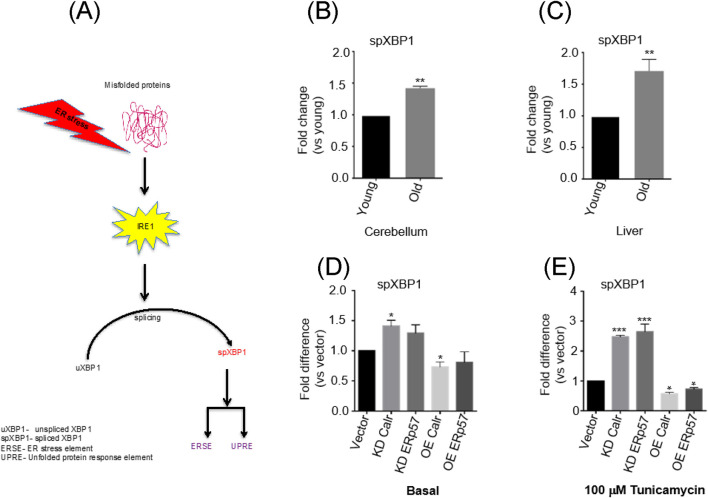
XBP1 splicing is prominent with age and is regulated by Calr and ERp57 expression. **(A)** Schematic representation between XBP1 splicing and ER stress. **(B)** mRNA levels of spliced-XBP1 (spXBP1) in the cerebellum and **(C)** liver mice tissues with age. **(D)** mRNA levels of spliced XBP1 in Calr and ERp57 RNAi or overexpression treated cells under the basal level. **(E)** mRNA levels of spliced XBP1 in Calr and ERp57 RNAi or overexpression of treated cells under stress conditions (100 µM tunicamycin). Data are expressed as the mean ± SD (n = 4). Means with the symbol (*) differ significantly; *p* < 0.05.

**FIGURE 6:**
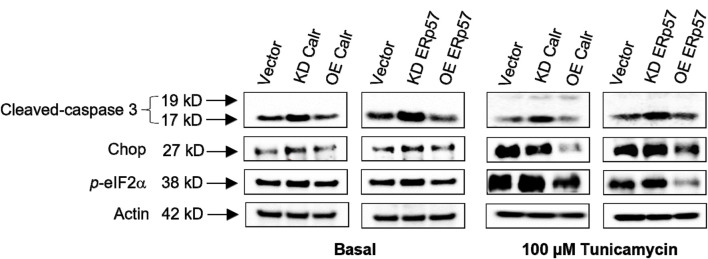
Calr and ERp57 regulate ER-stress-associated apoptotic signaling cascade. Western blot of ER stress markers cleaved-caspase 3, CHOP, and *p*-eIF2α under Calr and ERp57 RNAi or overexpression under basal conditions (left-side group of blots) and the same Western blots for the ER stress markers under stress conditions (right-side group of blots, +100 µM Tunicamycin). All blots are normalized by β-actin as the control. Each Western blot is a representative example of data from three replicate experiments. Data are expressed as the mean ± SD (n = 3). Means with the symbol (*) differ significantly; *p* < 0.05.

Upon induction of ER stress, the splicing of un-spliced X-box-binding protein 1 (uXBP1) mRNA to spXBP1 (spliced) leads to its frameshift, generating a strong transcription factor that can affect various genes involved in ER homeostasis, making XBP1 splicing a potent indicator of ER stress (Figure 5A; Yoshida et al., 2001). To gain insight into XBP1 splicing status during tissue aging, we measured the levels of the spliced form of XBP1 (spXBP1) in young and old cerebellum, as a representative brain regions, and in liver tissue. The mRNA level of spXBP1 was significantly increased in the mouse cerebellum (Figure 5B) and liver (Figure 5C) with age. It indicates that ER stress increases with age. In the cell model, we found that the spXBP1 mRNA level increased at the basal knockdown of Calr and ERp57 in N2A cells (Figure 5D); the application of the ER stressor tunicamycin further intensified XBP1 splicing in knockdown cells (Figure 5E). It indicates that reduced Calr and ERp57 expressions make the cells prone to ER-stress-induced splicing of XBP1. Additionally, overexpression of Calr and ERp57 in N2a cells showed a protective effect with respect to XBP1 splicing under both basal and stress conditions (Figures 5D,E), implying that cells with higher levels of Calr and ERp57 are more resistant to ER stress. Despite the well-organized network of ER chaperones that maintains ER homeostasis through efficient protein folding, some translocated proteins may fail to fold correctly, thereby increasing ER stress (McCaffrey and Braakman, 2016). Therefore, the reduction of Calr and ERp57 might be one of the initiating factors in XBP1 splicing by triggering its upstream signaling effectors. In the event of a strong ER strain, activation of the ER luminal major protein sensors, such as endoplasmic reticulum kinase (PERK), inositol requiring kinase enzyme 1 alpha (IRE1α), and activating transcription factor (ATF6), is initiated, and proteins in each of these sensors are connected with a cytosolic pathway, leading to apoptosis (Hiramatsu et al., 2015; Schönthal, 2012). We then sought to determine whether the reduction of Calr and ERp57 affects known ER stress pathway(s) that may provide further evidence of the broader impact of Calr and ERp57 reduction on the ER-stress-associated response system. To test this assumption, we measured the protein expression levels of ER-stress-associated apoptosis markers (cleaved-caspase 3, CHOP, and *p*-elF2α) by Western blotting analysis. There was a significant increase in cleaved-caspase 3 in Calr and ERp57 knockdown cells (Figure 6, left). Cleavage of caspase 3 is the cytosolic signal of perturbed ER equilibrium and one of the indicators of cell apoptosis (Siman et al., 2001). Additionally, the levels of CHOP, a stress-associated transcription factor, and *p*-elF2α, an ER-stress-associated protein homeostasis sensing factor, were also significantly increased in knockdown Calr and ERp57 cells. It validated our hypothesis that the reduction in these two proteins triggers an augmented ER-stress-signaling cascade (Figure 6, left). In contrast, cells overexpressing both Calr and ERp57 showed a strong downregulation of stress markers (cleaved-caspase 3, CHOP, and p-eIF2α), suggesting that overexpression of Calr and ERp57 reduces ER stress via the inhibition of the ER-stress downstream signaling cascade.

To assess the effect of Calr and ERp57 knockdown under stress conditions, we incubated Calr and ERp57 knockdown N2a cells with tunicamycin. Expectedly, a significant increase was observed in the cleaved-caspase 3 protein level. However, interestingly, reduced expression of Calr slightly decreased CHOP protein expression, while ERp57 expression reduction showed an increase in CHOP protein level (Figure 6, right). On the other hand, overexpression of Calr and ERp57 markedly reduced the expression of cleaved-caspase 3, CHOP, and *p*-elF2α (Figure 6, right), demonstrating that under stress conditions, the higher levels of Calr and ERp57 expressions were able to maintain ER homeostasis by constraining the downstream signaling process of the ER-stress-associated cascade.

### 3.7 Calr physically and functionally interacts with ERp57 while cooperating in ER stress

**FIGURE 7:**
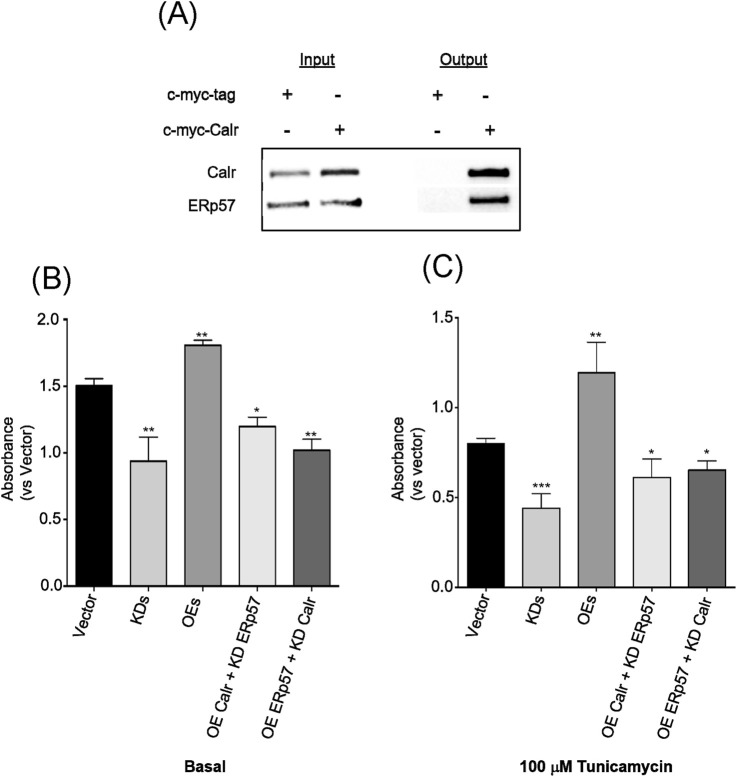
Calr physically and functionally interacted with ERp57. **(A)** Immunoprecipitation of Calr overexpressed N2a cells utilizing Myc-tagged beads. The resulting eluates were blotted with Calr, ERp57, and Myc-tagged antibodies. **(B)** Cellular viabilities of Calr and ERp57 co-transfections for both knockdown (KDs) and overexpressions (OEs) or combined co-transfection such as OE Calr + KD ERp57 and OE ERp57+ KD Calr under basal (left side) and **(C)** stress (right side, 100 µM tunicamycin) conditions, respectively. Each Western blot is a representative example of data from three replicate experiments. Data are expressed as the mean ± SD (n = 6) for the cellular viability assay and as ±SD (n = 3) for Western blot analysis. Means with the symbol (*) differ significantly; *p* < 0.05.

ER chaperones often interact with each other, both physically and functionally, to sustain ER homeostasis, and their activities are to intensify under conditions of ER stress. We tested whether Calr and ERp57 interact with each other, both physically and functionally, *in vitro*. First, to determine their physical interaction, a co-immunoprecipitation (Co-IP) assay was conducted using tagged Calr (c-myc-Calr)-overexpressing N2a cells. The results showed an anticipated physical interaction between Calr and ERp57, as shown in the detection of the ERp57 signal from the Calr protein output (Figure 7A). Then, we assessed the functional link between Calr and ERp57 by performing a simplified epistatic experiment with a concurrent cell viability assay under both basal and stress conditions. Co-transfection of the Calr knockdown plasmid with the ERp57 overexpression plasmid showed a drastic reduction in ERp57 overexpression-related cell viability, indicating that the activity of ERp57 is affected by Calr reduction. Similar results were obtained when an ERp57 knockdown plasmid was co-transfected with a Calr overexpression plasmid in terms of cell viability, further suggesting that Calr and ERp57 have an epistatic relationship. Furthermore, similar phenomena were observed under stress conditions (Figure 7C). The collected evidence from our study indicates that Calr and ERp57 work in tandem to ensure ER stability.

## 4 Discussion

The ER is a multipurpose organelle within which protein folding, post-translational modification, lipid biosynthesis, and calcium homeostasis occur. ER chaperones are evolutionarily conserved proteins that catalyze protein folding and post-translational processing across the animal kingdom. Age-associated decay of cellular apparatus leads to an increase in the incidence of protein unfolding/misfolding, accumulation, and aggregation, which is primarily due to the gradual deterioration of the chaperoning systems (Macario and Conway, 2002). Accumulated evidence indicates a role for unfolded/misfolded proteins in aging and age-related neurodegenerative diseases (Fargnoli et al., 1990; Hall et al., 2000; Pahlavani et al., 1995). The consideration of ER chaperones as biomarkers of aging is still debatable. This is partly because the expression of multiple ER chaperones does not follow the same trend with age, except for Calr and ERp57, which show a drastic decrease in protein levels with tissue aging (Figure 1A). Interestingly, a report suggested that Calr is downregulated in cortical neurons of Alzheimer’s disease (Cardoso et al., 2018). It is a possible indication of the biomarker potential of Calr and ERp57 in aging and age-related diseases.

As mentioned above, ER, aging, and age-associated diseases such as neurodegenerative disorders are interconnected in many aspects. The reduced expression of Calr and ERp57 with age may well preserve a functional purpose in the process of aging; therefore, we used the model organism *C. elegans*, with a shorter lifespan and straightforward genetic manipulations, to elucidate the potential functions/mechanisms of *crt-1* (Calr) and *pdi-3* (ERp57) in aging. The results were encouraging as the RNAi inhibition of *crt-1* and *pdi-3* significantly shortens the worm lifespan, indicating the pro-aging role of *crt-1* and *pdi-3* reduction. Additional studies, such as locomotion and stress resistance, will be needed to determine how the loss of *crt-1* and *pdi-3* affects additional aspects of organismal health during aging. In addition, both *crt-1* and *pdi-3* appear to intervene in the signaling components upstream of the key transcriptional activator DAF16 (FOXO) inside the cells of the insulin pathway in worm longevity. Further investigations using the stress-sensor strain of ER, SJ4005 (Arsenovicet al., 2012) worm, demonstrated a significant increase in ER stress when *crt-1* and *pdi-3* RNAi were applied. This implies that the shortened lifespan may be related to the increased ER stress in worms with reduced expression of *crt-1* and *pdi-3*. Intriguingly, previous studies in *C. elegans* have shown that mild or transient activation of the unfolded protein response can promote adaptive proteostasis and extend the lifespan (Henis-Korenblit et al., 2010; Taylor and Dillin, 2013). In contrast, chronic or unresolved ER stress is associated with loss of proteostasis, activation of pro-apoptotic pathways, and reduced organismal fitness (Walter and Ron, 2011). Our current data indicate that the significant reduction of *crt-1* and *pdi-3* resulted in a shortened lifespan, which might correspond to a failure to maintain ER proteostasis during aging rather than a hormetic ER-stress response. Moreover, future studies incorporating genetic rescue approaches will be crucial to further strengthen the mechanistic specificity.

Additional evidence was collected in the corresponding cell culture experimental setup by measuring the spXBP-1 mRNA, an indicator of ER stress (Hirota et al., 2006), and the results proved that ER stress increased considerably after knockdown of Calr and/or ERp57. Moreover, the marked increase in sp-XBP1 mRNA observed in the cerebellum and liver of aged mouse tissues in this study indicates that XBP1 mRNA splicing may also serve as a molecular marker of the aging process. This finding is in consistent with previous reports regarding the increase in XBP1 splicing with age (Yin et al., 2010). Notably, the level of spXBP-1 mRNA was negatively correlated with the expression level of Calr and ERp57 *in vitro*. This is a new line of evidence showing the reduction of Calr and ERp57 as a possible initiating factor for XBP1 splicing.

Aberrance in the ER protein folding system can inevitably trigger the accumulation of unfolded/misfolded proteins that induce ER stress. Whether the reduction of Calr and ERp57 prompts the buildup of misfolded protein is yet to be elucidated. Remarkably, knockdown of both Calr and ERp57 in N2a cells showed an increase in the accumulation of misfolded proteins, reflected as protein aggregation, and a decrease in cell viability. Cell death induced by ER stress is associated with the activation of a pro-apoptotic signaling cascade. Evolutionarily conserved ER-stress-sensing cascades, such as UPR and ERAD, play a critical role in limiting the burden of misfolded proteins and enhancing protein-folding efficiency by inducing the transcription of chaperone genes, which increase ER folding capacity, and by promoting the translocation of misfolded proteins to the cytosol for degradation (Ni and Lee, 2007). They differ in signaling transductions, but they all converge into cell apoptosis. Reduced expression or function of Calr and ERp57 with age exacerbates ER-stress-related protein aggregation, thereby compromising cell viability and organismal lifespan.

Cell intrinsic signaling initially responds to ER stress in ways that help maintain cellular homeostasis. For example, inhibiting the global translation during the sustained ER stress by activating the translation initiation factor (eIF2α), a major cytosolic ER stress effector, provides ample time for the ER to accommodate the clearance of misfolded proteins (Brown and Naidoo, 2012). Our study showed that the expression of *p*-eIF2α under both basal and stress conditions was increased in Calr and ERp57 knockdown cells, whereas it was reduced in cells overexpressing Calr and ERp57. It indicates that the reduction in Calr and ERp57 expression triggers the ER-stress-related translational perturbation. Meanwhile, the ER-stress-induced *p*-elF2α and spXBP-1 will further activate the downstream potent pro-apoptotic components, such as CHOP and the cleavage of caspase 3 (cleaved caspase-3), and, thus, promote cell apoptosis. Basal and inducible CHOP increase with age, and further induction of its expression was observed in stressed aged rats but not in their younger counterparts, indicating that aged animals are susceptible to apoptosis (Paz Gavilan et al., 2006; Kirkland et al., 2002; Ikeyama et al., 2003). CHOP and cleaved caspase-3 were elevated in Calr and ERp57 knockdown cells. However, under stress conditions, the results varied slightly: an increased expression of CHOP was evident in ERp57 knockdown cells, whereas a slight decrease was detected in Calr knockdown cells. It indicates that Calr and ERp57 may slightly differ in response to further increases in ER stress. This observation is also reflected in our worm results, where *pdi-3* RNAi showed more induction of ER stress than that of *crt-1*. Findings from our study also demonstrated a substantial reduction in cleaved caspase-3, CHOP, and *p*-elF2α expressions under both basal and stress conditions with higher level of Calr and ERp57. This scenario is consistent with the previous reports regarding the overexpression of Calr and ERp57 as restorative elements in protein misfolding-induced ER stress in neurodegenerative and immunity-related diseases (Hoshino et al., 2007; Hetz et al., 2005; Hetz and Soto, 2006). Although the abovementioned ER-stress markers are widely used and sufficient to support the current study of ER-stress observed phenotypes, analysis of ATF6 activation, ERAD flux, and proteasome involvement would be of great significance for providing additional mechanistic details and important directions for future investigation.

Most ER resident chaperones form a complex to process the fragile nascent proteins for ensuring proper folding and maturation (Tatu and Helenius, 1997; Jansen et al., 2012). The interaction/dependency of Calr and ERp57, which affect the efficiency of their individual functions in the ER, remain elusive, although reports have shown that ERp57 and Calr depend on each other during co-translocation to the plasma membrane in cancer treatment (Panaretakis et al., 2008). Our study provides evidence that both Calr and ERp57 are physically and functionally interconnected and play a role in ER-stress-associated cell survival and organismal longevity. The increase in ER stress along with the aging process has been well-documented (López-Otín et al., 2013). However, understanding of the decline of specific ER chaperones and their physiological consequences with age remains limited. We present emerging evidence of the ubiquitous reduction of Calr and ERp57 in tissue aging, along with the possibility that this reduction may contribute to compromised cell viability and a shortened lifespan through increased ER stress. Our study indicates that the disruption of ER homeostasis could not only be a hallmark of aging but also a factor that accelerates the aging process. To the best of our knowledge, there is no direct evidence yet linking the combined activities of Calr and ERp57 to ER-stress-induced aging. Although a report showed that Calr and ERp57 are bound to β-amyloid in the cerebrospinal fluid of patients with Alzheimer’s disease (Erickson et al., 2005), the critical role of Calr and ERp57 in the ER in age-associated diseases such as Alzheimer’s disease requires further elucidation.

## Data Availability

The original contributions presented in the study are included in the article/Supplementary Material, further inquiries can be directed to the corresponding author.
