# Supplementary material for: Reduction of calreticulin and ERp57 with age reveals the ER stress-related roles in cell viability and organismal lifespan regulation
Source: Front Aging. 2026 Feb 19;7:1758247. doi: 10.3389/fragi.2026.1758247 (PMC12960552; doi:10.3389/fragi.2026.1758247)
Supplement: Supplementary file 1 [file DataSheet1.docx]

Supplementary Figures for” Calreticulin and ERp57 Reduction with Age Reveals the ER Stress-related roles in Cell Viability and Organismal Lifespan Regulation”

**Gregor Burdeos^1,2,*^, Sophie Neuber- Schlicht^1,3^**

^1^Max Planck Institute for Biology of Ageing, 50931 Cologne, Germany

^2^Research Institute for Farm Animal Biology (FBN), Wilhelm-Stahl-Allee 2, 18196 Dummerstorf, Germany

^3^Department of Chemistry Bioorganic Synthesis, Humboldt-Universität zu Berlin, Brook-Taylor-Str. 2,12489 Berlin, Germany

*** Correspondence:**Corresponding Author: Gregor Burdeos
burdeos@fbn-dummerstorf.de

**
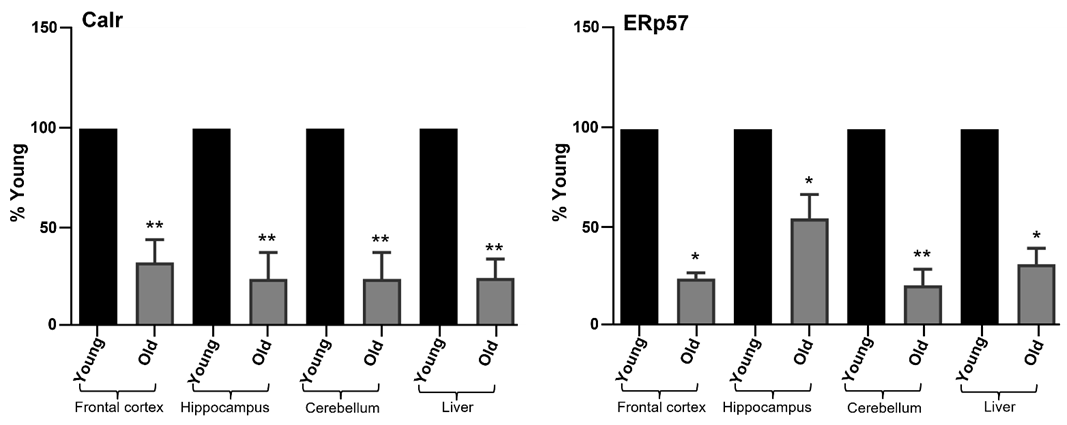
**

**Figure S1.** Protein qualifications of both Calr and ERp57 in young and old mice brain regions and liver. Each Western blot is a representative example of data from three replicate experiments. Data are expressed as mean ± SD (n = 3). Means with symbol (*) differ significantly, *P* < 0.05.

(B)

(A)


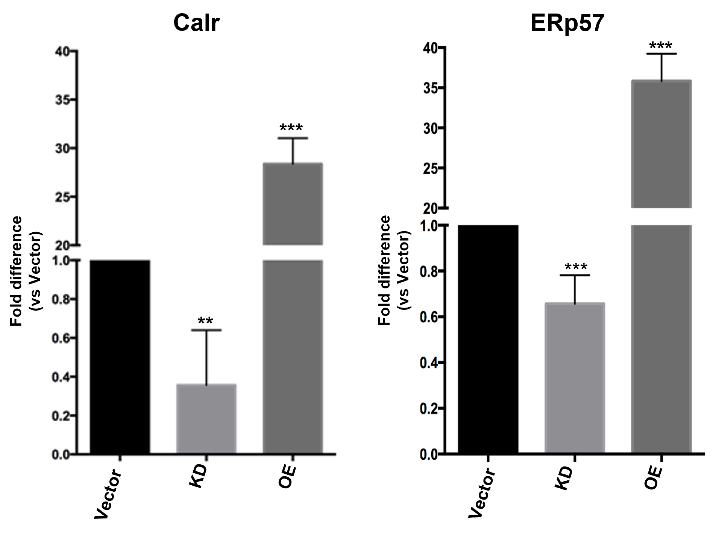


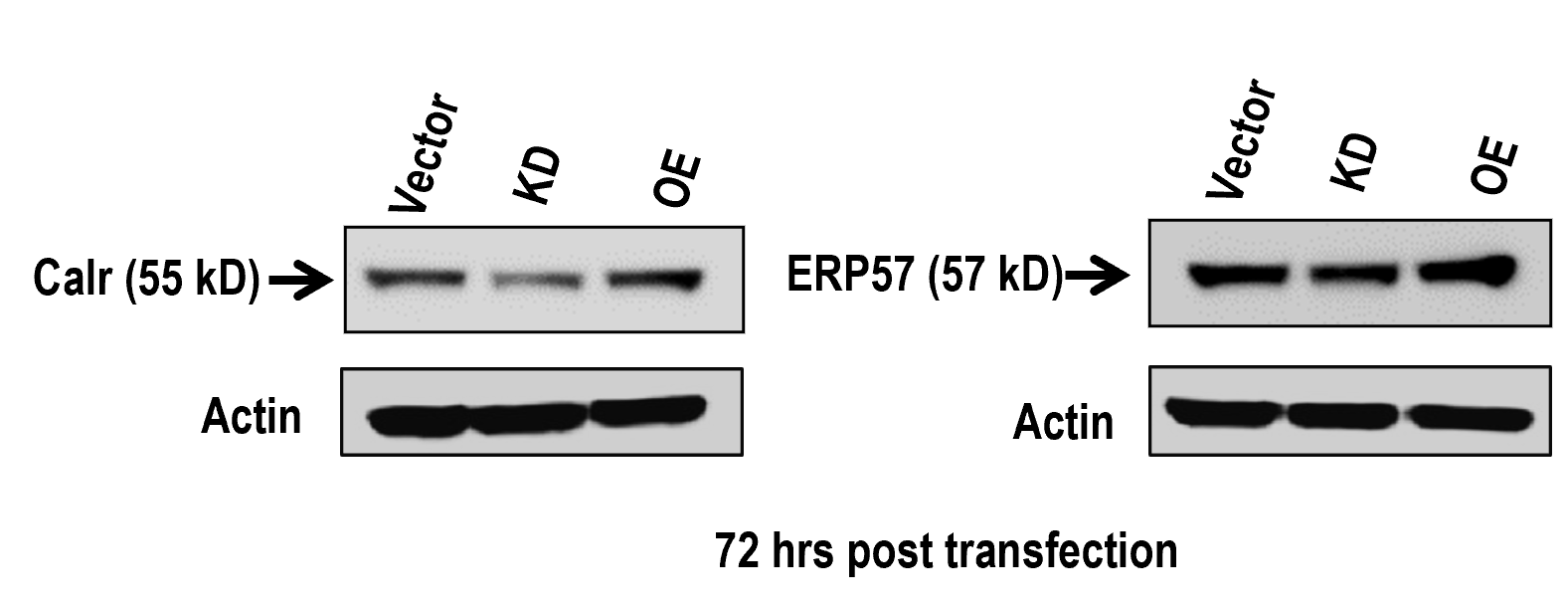


(D)

(C)


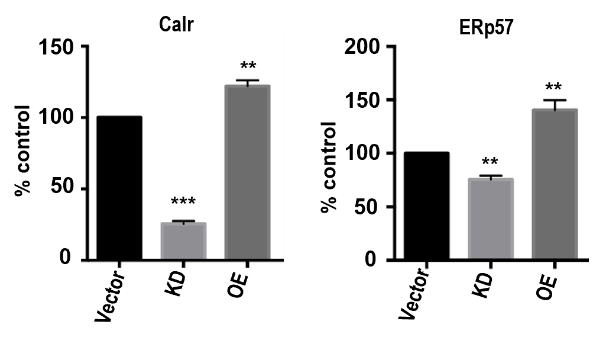

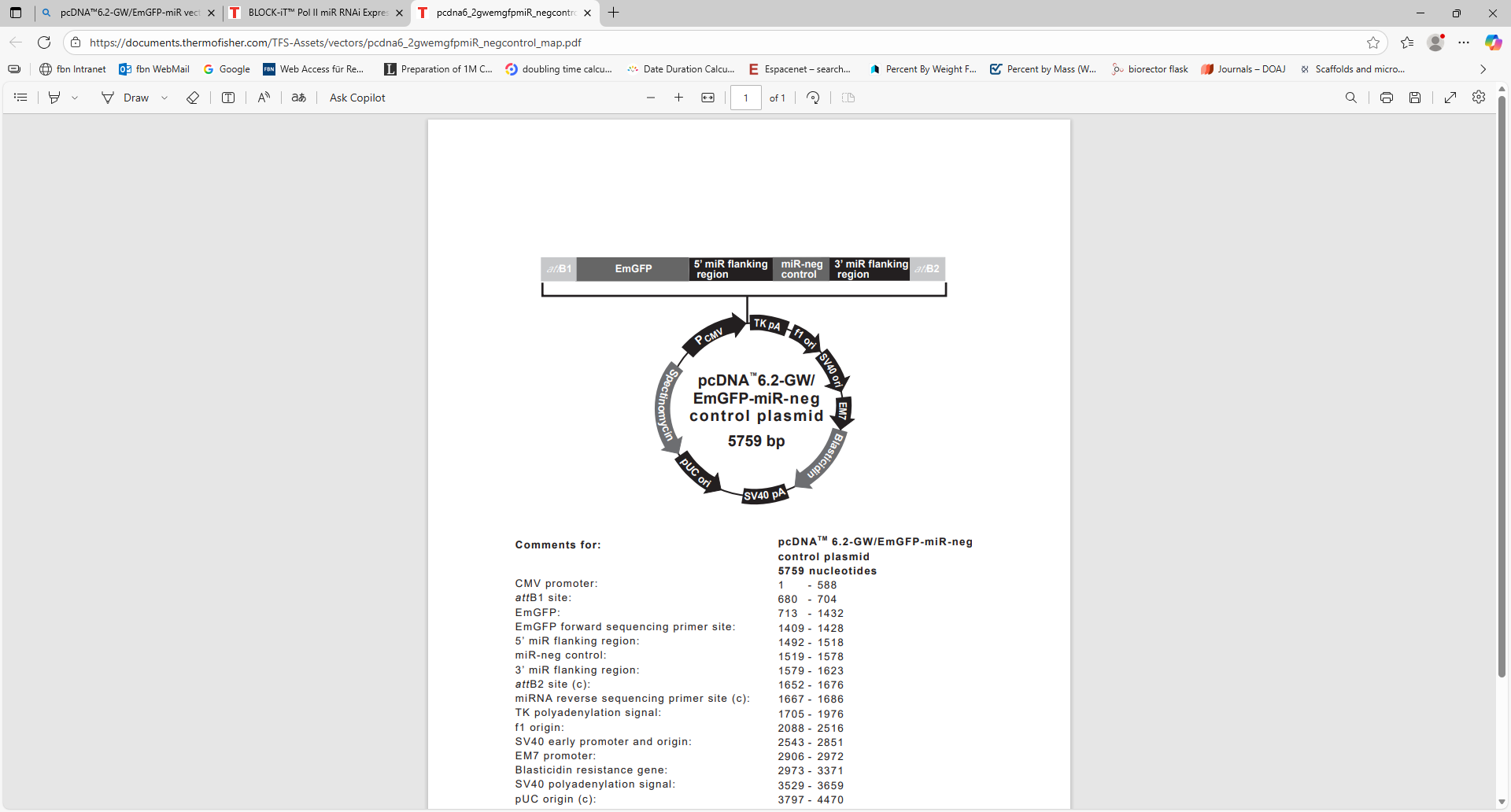


(E)


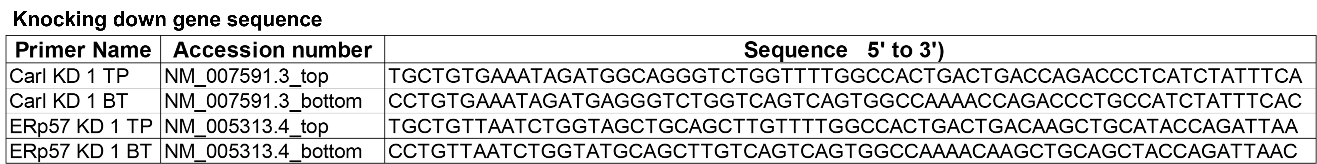


(G)

(F)


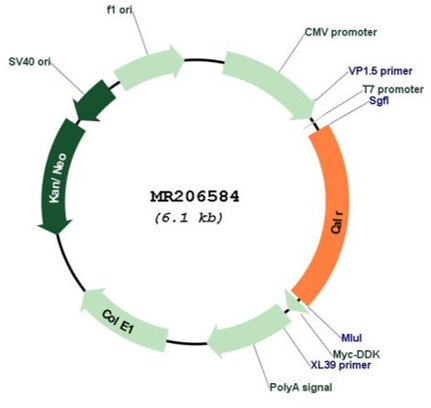

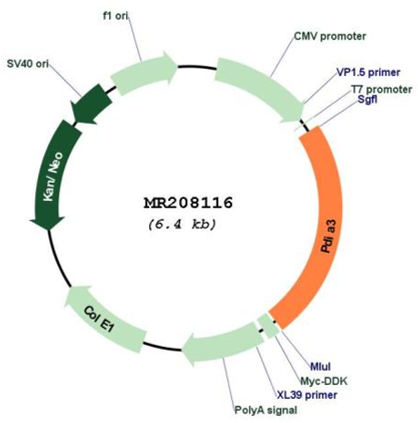


(H)


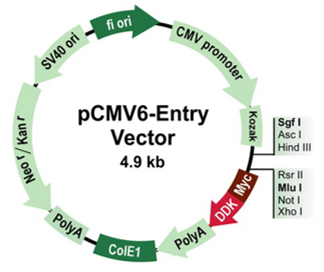


**Figure S2.** Genetic manipulation of N2a cells. (**A**) mRNA expression levels qualifications of Calr and ERp57 after siRNA (knocking down) and overexpression 72 hours post transfection. (**B, C**) Immunoblot and its quantifications of knocked down and overexpressed Calr and ERp57 after 72 hours post transfection. (**D, E**) Vector map (pcDNA™6.2-GW/EmGFP-miR vector) and primer sequences for siRNA of Calr and ERp57. (**F, G, H**) Overexpression vector map of Calr and ERp 57 (pCMV6 entry, c-Myc tagged-Calr) and ERp57 (pCMV6 entry, c-Myc tagged-Pdia3). Data are expressed as mean ± SD (n = 4 for mRNA and n=3 for protein qualifications). Means with symbol (*) differ significantly, *P* < 0.05.
